# The Influence of Gamification and Information Technology Identity on Postadoption Behaviors of Health and Fitness App Users: Empirical Study in the United States

**DOI:** 10.2196/28282

**Published:** 2021-07-05

**Authors:** Pouyan Esmaeilzadeh

**Affiliations:** 1 Department of Information Systems and Business Analytics College of Business Florida International University Miami, FL United States

**Keywords:** gamification, health and fitness apps, IT identity, continued intention to use, information-sharing tendency, mHealth, app design, user interaction

## Abstract

**Background:**

The use of health and fitness apps has been on the rise to monitor personal fitness and health parameters. However, recent research discovered that many users discontinue using these apps after only a few months. Gamification has been suggested as a technique to increase users’ interactions with apps. Nevertheless, it is still not clear how gamification mechanisms encourage continued use and inspire user self-management.

**Objective:**

The main objective of this study was to articulate how gamification mechanisms in studies of designing and using health and fitness apps can contribute to the realization of information technology (IT) identity and positive behavioral outcomes. The broader goal was to shed light on how gamification mechanisms will translate into positive use behaviors in the context of mobile health apps.

**Methods:**

Data were collected from 364 users of health and fitness apps through an online survey to empirically examine the proposed model.

**Results:**

Based on identity theories, this study suggests the fully mediating role of IT identity to describe how gamification elements can lead to continued intention to use health and fitness apps, and increase users’ tendency for information sharing through the apps. The findings indicate that perceived gamification can increase users’ IT identity. In turn, a higher IT identity would encourage users to continue using the apps and share more personal health information with others through the apps.

**Conclusions:**

The results of this study can have practical implications for app designers to use gamification elements to increase users’ dependency, relatedness, and emotional energy associated with health apps. Moreover, the findings can have theoretical contributions for researchers to help better articulate the process in which gamification can be translated into positive use behaviors.

## Introduction

### Mobile Health

In recent years, many companies have invested in developing mobile health (mHealth) apps. The concept of mHealth is defined as a medical and public health practice supported by mobile devices and applications [1], including using a mobile app to monitor a patient’s blood pressure remotely or using an app to monitor, control, and track personal health data for fitness or diet purposes. mHealth provides an alternative to improve communication, care delivery, real-time medication monitoring, and adherence support [2]. mHealth technologies have the potential to significantly impact health care and health outcomes [3]. The increasing adoption of smart devices and the development of health and fitness apps have enlarged the growth of the mHealth market. Despite the large number of health care apps developed, the majority have encountered issues of underutilization and decline in user eagerness [4].

### Gamification in mHealth

Different health-related apps have attempted to leverage various techniques to attract and retain more users and enhance user interactions. One of the frequently used methods is creating a gameful design for health app services [5]. Gamification in services is defined as “a process of enhancing a service with affordances for gameful experiences in order to support user’s overall value creation” [6]. Structural gamification uses game elements to encourage users to attain a goal. Structural gamification does not modify the content of a process but instead changes the structure of that process [7]. Gamified elements used in mHealth are important to enable user self-management. Gamification mechanisms in developing health and fitness apps include badges, leaderboards, points and levels, challenges and quests, as well as social engagement [8]. For instance, badges are used to identify individual achievements in accomplishing goals, whether relative or nonrelative to other peers [9]. There are many health apps with gamification mechanisms currently available, such as mySugr with a challenges mechanism, RunKeeper with leaderboards and social engagement elements, and Fitocracy with badges.

As the mHealth market size is booming, gamification is now recognized as an influential factor affecting self-engagement in care management. However, approximately 80% of apps that use gamified elements will fail due to the poor design of gamification mechanisms [10]. Gamification is an engaging mechanism of video games used in nonvideo game contexts [11]. However, game design elements would not lead to more engagement or continued intention to use health apps if they cannot trigger users’ dependency, relatedness, and emotional energy associated with the apps. Previous studies indicate that using gamification elements may not be sufficiently exciting for users to continue using the apps without further motivation and reinforcement [12]. This is why many users discontinue using health-related apps (even gamified apps) after only a few months [13]. Thus, gamification mechanisms may encourage people to interact with the apps; however, they cannot necessarily guarantee continuous engagement of users with their health apps to control their health status or check personal health information.

Using self-management tools to monitor health information is consistent with the premises of patient-centered health models, which consider more control and responsibilities for patients as an important stakeholder in the health care ecosystem [14]. Nevertheless, little is known about how gamification mechanisms can motivate people to actively use health and fitness apps, and share their personal health information through such apps [15]. Thus, further research is required to investigate users’ beliefs and perceptions of their health apps to uncover the missing link between gamification and positive use behaviors. To fill this gap, the concept of information technology (IT) identity was adopted as a theoretical basis for this study to describe how gamified health apps can result in increased use behaviors and improved information sharing through apps.

### IT Identity

Previous studies have examined the topic of IT and identity and their relationship using different approaches [16]. For instance, one method treats IT as part of self-perception and identity to explain who people are in relation to IT. This approach proposes the possibility of viewing IT as an integral part of the self. One study highlighted that technology could be an essential part of people’s identities if they are emotionally attached to it [17]. IT identity is conceptualized using theories on social structures and self-concept to describe how people categorize themselves in relation to an IT object [18]. Thus, IT identity is defined as the extent to which the use of IT is saliently related to who people think they are (self-identification) [19]. IT can affect people’s self-perceptions by activating their original selves in using IT’s capabilities and resources. In turn, using IT frequently, individuals may feel empowered, creative, independent, and accessible. Through gamification elements such as points, leaderboards, and challenges, gamified health apps can be integrated into a user’s sense of self as they may spend a significant portion of their time interacting with the apps as a repeated behavior. This point is consistent with previous studies proposing that repeated behaviors may directly lead to identity recognition if such interactions initiate emotion, dependence, and affiliation [20]. In the context of gamification, individuals may use gamified health and fitness apps to become autonomous in controlling their fitness and wellness, monitoring physical changes, and managing emergency cases. These functionalities may enrich users’ original self-perceptions and make them feel autonomous, empowered, and capable. Thus, gamification elements may elevate users’ IT identity, and inspire them to continue using the apps and share more health information with other users through gamified features.

### Objectives and Research Questions

The main objective of this study was to examine how gamification mechanisms in studies of designing and using health and fitness apps can contribute to the realization of IT identity and positive behavioral outcomes. IT identity may provide a possible foundation for answering questions about how individuals become more likely to continue using the apps and share their personal health information with others using a gamified health app. The main hypothesis of the study is that gamified health apps will lead to positive use behaviors only when a strong IT identity related to the apps is activated. Consistent with the study objective, a research model was developed by drawing on the recent appearance of the IT identity concept in the information systems literature, which was adapted for using health and fitness apps. In short, this study addresses the following questions: (1) Can gamification mechanisms influence users’ continued intention to use apps and their information-sharing tendency? (2) Does IT identity fully mediate the relationship between perceived gamification and positive use behaviors?

### Literature Review

#### Gamification Mechanisms in Health and Fitness Apps

According to Werbach [21], gamification is the process of making activities more game-like. Thus, a combination of components that constitute games can result in the holistic experience of gratefulness. Robson et al [12] define a framework consisting of three gamification principles—mechanics, dynamics, and emotions—to explain how gamified experiences can be created. Previous studies highlight that gamification can be used in different contexts to affect individuals’ behaviors or attitudes [22].

Gamification is composed of two subcategories: structural and content gamification. Using structural gamification, designers leverage some game elements (such as digital badges and leaderboards) to encourage users to achieve a goal [23]. Content gamification modifies content to make it more game-like. An example of this type of gamification would be adding story, challenge, curiosity, mystery, and characters to change content to engage the learner [24]. There are different gamification mechanics; however, the commonly used game elements in mHealth apps can be categorized into five main groups: badges, leaderboards, points and levels, challenges and requests, as well as social engagement loops and onboarding [10].

Badges are used to identify and reward individual achievements. Users can achieve badges by completing the task described. Gamified apps use a dashboard to provide a summary of all badges obtained by users. Digital badges enable users to visualize their performance and review their personal progress [25]. For instance, users of a fitness tracker app should enter some details (eg, weight lifted or distance run) to pass a threshold value and achieve a badge. Leaderboards mainly rank individual user progress and achievements as compared to their peers. Users understand what constitutes their position on the leaderboard and what actions could be taken to raise the leaderboard compared with their peers [26]. For instance, users of a fitness tracker app may see how they are ranked compared to their peers in the context of their local and global networks.

Points and leveling systems are implemented to inform the user of their level of familiarity, and reward continued expertise and knowledge using the system. Progress bars are also a standard feature of points and levels, where users can monitor how many points they have already attained along a continuum and how many more points they need to obtain to move up to the next level [27]. For instance, users of a nutritional supplement manager app will advance to higher levels by achieving points. This process is reinforced by tangible rewards in cash and the intrinsic reward of effectively using the app to manage their regimen. Continued challenges and quests may motivate users to interact with the app frequently, especially where these challenges confirm their understanding of the app’s goals [10]. For example, users with diabetes may complete daily challenges, and an avatar provides feedback to them concerning whether they have taken satisfactory steps in managing their diabetes for the day. Finally, social engagement loops enable information sharing between app users. The integration of social media platforms such as Facebook and Instagram into health and fitness apps can increase user enjoyment and engagement through personal information sharing [10]. Users may want to share health-related data and personal achievements from their apps to nurture social capital with other users and gain support from these peers. The social loops also facilitate onboarding in which new users can join the network via invites from existing users. For instance, users of a fitness tracker app will have an option to register through their Facebook or Twitter accounts. The “friends” tab then enables users to find their friends and add them to their personalized fitness network. Thus, users can exchange fitness challenges with their peers, and track and post their progress on social media.

#### IT Identity in the Context of Health and Fitness Apps

Previous studies suggest that using IT can expand the actual self along with the meanings and insights related to the self [28]. By incorporating health and fitness apps into routine health-related activities, achieving personal goals and challenges (such as diet, nutrition, and heart rate) will be significantly dependent on using the apps [29]. Technology’s functionalities and capacity can amplify individuals’ personal and social resources in attaining their goals [30]. Health and fitness apps allow users to expand the self by offering valuable resources and capabilities that help them accomplish self-management goals. As mentioned in previous research, identities can be altered through interventions such as interactions with IT [31]. IT identity can be actualized when individuals frequently engage with technology as an end user. Some technologies are more likely to foster IT identity than others because users may consider them an inherent part of themselves, affecting their behavioral choices. For instance, health and fitness apps are consumer IT that can become part of users’ identities due to their routine use for self-monitoring and self-management purposes [32]. As individuals frequently interact with different features of health and fitness apps to check their health status, the apps provide self-confirming feedback to users, which in turn nurtures their IT identity.

IT identity represents the extent to which the use of a target IT contributes to the sense of self and self-identification [18]. IT identity associated with a target IT is reflected in users’ emotional reactions (emotional energy, relatedness, and dependence) to thinking about themselves in relation to the IT with which they interact. Thus, IT identity consists of three subdimensions: relatedness, dependence, and emotional energy attached to IT [18]. Relatedness refers to a sense of connection felt when interacting with a target IT. Users’ perception of the self and what they can do with a target IT is a function of a strong connection with the IT [33]. For example, when individuals with stronger IT identity think of themselves in relation to health and fitness apps, they see themselves as linked with the apps. Emotional energy refers to the emotional attachment, passion, and self-assurance that users associate with a target IT when thinking about their interaction with it. Enduring user-IT engagement can elevate the user’s emotion and confidence with the IT, and allow the user to be more spontaneous with the technology [34]. For instance, when individuals with formed IT identity think of themselves with their health and fitness apps, they feel enduring enthusiasm about the apps. The dependence dimension describes how individuals rely on a target IT to characterize their self-perceptions. For instance, people may need to rely on digital communication platforms to manage their relationships with others and meet social expectations [35]. When individuals with activated IT identity think of themselves with health and fitness apps, they feel that they can count on the apps to monitor their health and fitness data.

### Hypotheses Development

#### Gamification and IT Identity

The main proposal of this study is that gamified features of health and fitness apps influence individuals’ self-identification with a target technology. Theory on IT identity suggests that technologies with broad application across social situations (eg, mobile devices and software applications) are most highly expected to enact IT identity [18]. Gamification mechanisms are mainly applied to health and fitness apps to better engage users to monitor their health status and check their personal information. On this basis, the gamified elements can influence individuals’ IT identity via two means: (1) giving more control over the apps through gamified and customizable features and (2) intrinsic benefits that foster emotional responses in relation to the apps.

There is evidence to support that self-identification of individuals with material objects that they can manipulate are more likely to be activated than with those they are less likely to exercise control over [19]. Interactions with the gamified features of apps result in feelings of mastery, competence, and a belief in one’s ability to control health-related tasks (such as health status monitoring). For instance, individuals who can choose to achieve specific digital badges, participate in customized challenges, and share their data on selected social platforms are more likely to identify with the app than those who cannot. A fitness app provides users with a personalized meal plan and workout plan consistent with a chosen body goal. Thus, when an individual connects gamified elements while exercising control over an app, the subsequent feelings of competency and passion will create a sense of adapting to the app’s gamified feature set. Consequently, this evaluation positively shapes the user’s self-identification with the technology.

Previous research indicates that individuals need some degree of internal gratification to initiate and continue a job [36]. People are more likely to activate identities that generate more intrinsic enjoyment and satisfaction than those with few material benefits [37]. According to identity theories, the level of benefits (such as emotional attachment) offered by a target IT could foster positive self-identification with the IT [38]. Gamified apps can build emotional responses through increasing enthusiasm, dependence, and affiliation in relation to the apps. Net benefits (such as energy, joy, and connection) that users associate with use of an IT can motivate users to identify the self with the IT [39]. Previous studies highlight that the intrinsic benefits positively impact emotional responses to an IT through the sense of reliance and dependence on the IT [40]. For example, rewards and incentives (such as points and badges) offered by health and fitness apps will lead to more engagement and connection with the apps. Gamified features motivate users to complete the task described to achieve their personal goals and gain defined rewards (eg, reaching better positions relative to others in the ranking system). Gamification mechanisms incentivize users to earn inherent benefits (such as informational and emotional support) by sharing personal data on social engagement loops. Moreover, people are more likely to consider IT an integral part of their self-concept when interactions with IT can improve their desired self-image [41]. For example, a gamified app will provide useful feedback to users to take adequate steps and attain their preferred self-concept. Therefore, the first hypothesis is as follows:

H1: Perceived gamification mechanisms used in health and fitness apps positively influence users’ IT identity.

#### IT Identity and Positive Use Behaviors

Recent studies recognize a need for alternative theoretical perspectives to examine technological and individual factors in the postadoption stage of using IT [13]. For instance, the concept of IT identity, which reflects positive self-identification using familiar technology, can encourage positive IT usage [19]. IT identity offers a richer understanding of users’ postadoption interactions with IT. For instance, users with a strong IT identity are more likely to explore different IT features and use them to complete additional tasks [18]. In the context of personal health devices, a recent study showed that IT identity is positively associated with feature use behavior and enhanced use behavior [15]. On the same basis, individuals’ emotional responses to themselves concerning health and fitness apps may motivate them to continue using the apps. Users with a stronger IT identity are more dependent, related, and emotionally connected to the apps they are using. Thus, this strong sense of connection and enthusiasm they attach to the apps inspire them to keep up with the apps.

IT identity affects how people use the various features of personal health devices in different situations [15]. When individuals consider a health and fitness app an integral part of the self, they may become more likely to continue using a significant number of its features in the future. Higher levels of emotional energy, confidence, and dependency attached to the apps may motivate users to continue interacting with the apps and explore new functionalities. Users with an activated IT identity tied to health and fitness apps would desire to carry on with its features to achieve multiple health-related purposes. For example, they may continue with the apps to track activity, diet data, calories burned, and control fitness as well as wellness data [42]. Therefore, the second hypothesis was framed as follows:

H2: IT identity attached to health and fitness apps positively influences continued intention to use the apps.

A feeling of dependency and enthusiasm in relation to mHealth apps can result in continuous, effective, and long-term use [16]. One line of evidence supporting the effective use of mHealth apps is their application to disclose health information in the hope of receiving more accurate feedback [43]. Users with a strong IT identity are more likely to develop autonomy to use interactive apps; thus, they become more open to deliberately share further personal health information to complete additional health-related tasks. A sense of connection and dependence on an app may assure users to reveal more health data to better accomplish fitness and wellness goals. Moreover, they may share personal information to seek new ways of using the apps in other situations to fulfill the optimal self-identity expression [44].

Positive emotional energy between users and a health app may empower them to discover previously unused features [18]. Using feature extensions may indicate sharing more personal information, and allowing the apps to access their data to participate in more challenges and obtain extra points. For instance, exploring an underutilized set of features may require users to frequently report their health status every week. The sense of connection and enthusiasm may give more confidence to users to disclose more data for performing tasks such as monitoring and checking health measures. Thus, individuals with a stronger IT identity may desire to verify themselves by sharing personal data to use broader features of a health and fitness app.

IT identity can stimulate users to explore more resources and capabilities offered by the health and fitness apps. For example, IT identity holders who were unaware of remote care and medical surveillance afforded by a health and fitness app will attempt to reveal more personal data to explore these capabilities and add these services to their to-do list. Thus, attraction toward the app may encourage individuals to share more personal data to find new situations to apply the apps in the daily roles they maintain. Individuals with a strong IT identity may actively involve collecting more facts about an IT’s features to use them for additional tasks [45]. Holders of IT identity may search for more information, educate themselves about the apps’ features and requirements, and share more personal data to use new and existing features to perform various health-related tasks, leading to the following hypothesis:

H3: IT identity attached to health and fitness apps positively influences information-sharing tendency.

#### Mediating Role of IT Identity

The related literature indicates that gamified features of mHealth apps are necessary but not sufficient to motivate users to keep up with the apps or encourage them to share their personal health information [46]. Applying various gamification mechanisms to health and fitness apps is insufficient because they alone cannot shape postadoption behaviors (such as information sharing and continued intention to use the apps). Previous studies demonstrate that users will not continue using gamified apps or are not likely to disclose their health information simply because they have gamified designs [47]. On the same basis, gamification mechanisms may result in positive use behavior only if the gamified design first fosters IT identity. The activated IT identity in relation to the gamified apps will then lead to positive postadoption behaviors. Thus, gameful and playful designs should first form the feeling of emotional energy, dependence, and relatedness in relation to the apps, and then in return intensify intention to stay with the apps and share more personal health information via the apps. This proposal is formally hypothesized as:

H4a: IT identity attached to health and fitness apps fully mediates the relationships between perceived gamification mechanisms and continued intention to use them.

H4b: IT identity attached to health and fitness apps fully mediates the relationships between perceived gamification mechanisms and information-sharing tendency.

### Research Model

**Figure 1:**
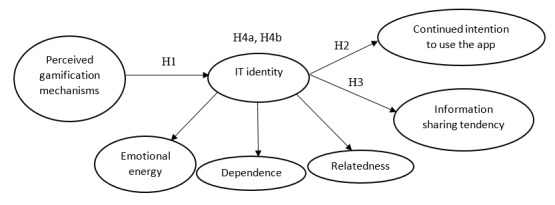
Research model. IT: information technology.

According to the theoretical rationale for the proposed causal relationships, the research model presented in Figure 1 was developed to guide this study. This research model suggests a framework to examine the effects of perceived gamification on positive use behaviors. In particular, the model proposes that perceived gamification mechanisms used in health and fitness apps directly foster IT identity, which positively shapes continued intention to use the apps and information disclosure willingness. The model further posits that IT identity in relation to the apps fully mediates the relationship between perceived gamification and positive use behaviors.

It should be mentioned that, consistent with prior studies, IT identity is considered as a second-order construct with three reflective factors [48]. The rationale behind this measurement is that IT identity is reflective of the three dimensions (ie, relatedness, emotional energy, and dependence) and the expected interactions among them. Therefore, all of these dimensions can reflect the same theme and may covary. According to Kayhan [49], reflective modeling is a better option than formative modeling when first-order factors are expected to interact, correlate, or share a common theme. Thus, a set of interrelationships among these three factors is an essential component of measuring IT identity.

## Methods

### Research Approach

To achieve this study’s objective, an online survey was administered to examine the proposed relationships between gamification mechanisms, IT identity, and positive use behaviors. First, a scenario was designed about why people are attached to their health and fitness apps by describing the “IT identity” and “gamification” concepts. One screening question limited the participants to those who used a gamified health app to monitor, control, and track health data for physical activities, weight loss, fitness, or diet purposes. Respondents were then asked to fill out the survey considering one health or fitness app (with gamification aspects) that they used. For instance, when a respondent named MyFitnessPal as the app that they used, they would answer all questions with consideration of that particular app. The logic behind this filter was to measure perceived gamification mechanisms and IT identity in relation to a target IT (ie, a specific mHealth app). This enabled examining the roles of gamification and IT identity in shaping positive use behavior (ie, continued use and information-sharing tendency) associated with that particular app.

Moreover, respondents were asked to describe how long and how often they used the app and to what extent they were familiar with the particular app they mentioned. In the second section of the survey, respondents were asked to think about the scenario that they read in the survey, and express their perceptions about the gamification mechanism designed for the app, IT identity in relation to the app, continued intention to use the app, and willingness to share information with the app. The next section of the survey was dedicated to demographic variables and general IT experience questions.

### Survey Development

The approach used to measure the variables of the research model was based on existing literature. The survey items were adapted from previously validated surveys with slight changes made to fit the context of this study. The description of the proposed variables and the sources used to develop the questionnaire are provided in Table 1. The measure items (ie, all the questions included in the survey) are listed in Multimedia Appendix 1.

**Table 1:** Operationalization of constructs.

Construct	Construct definition	Source
Perceived gamification mechanism	The extent to which an app uses gamification elements (such as badges, leaderboards, points, challenges, and social engagement) in its design	Miller et al [10]
IT^a^ identity	“The extent to which an individual views use of an IT as integral to his or her sense of self”	Carter and Grover [18]
Continued intention to use the app	The extent to which an individual is willing to continue using an app	Karahanna et al [50]
Information-sharing tendency	The extent to which an individual is likely to disclose his/her personal health information through an app	Bansal and Gefen [51]

^a^IT: information technology.

### Pilot Test

Once the initial questionnaire was developed, six professionals in the health app domain were consulted to improve the content validity of the study, and finalize the gamification mechanisms and the questions used in this study. Consistent with the experts’ suggestions, the terms used to define gamification were modified, and the scenario and questions were improved to ensure that they were sufficiently transparent and easy to understand for the public. Face validity was then performed with 23 students (6 PhD candidates, 7 with Master’s degrees in information systems, and 10 undergraduate students) to ensure that the readability and wording of the questions were acceptable and consistent with the objectives of the study. Thus, some ambiguous terms were reworded, and technical language and jargon were removed so as to describe the scenario in the most understandable way. Finally, prior to the main data collection, a pilot test was performed with 152 undergraduate students at a large university in the southeastern United States to ensure that the instrument had acceptable reliability and validity. The Cronbach α was computed for each construct (ie, perceived gamification mechanism α=.91, IT identity α=.90, continued intention to use the app α=.94, and information-sharing tendency α=.90). All Cronbach α values were above the cut-off point of .70, indicating that the instrument was internally consistent [52].

### Data Collection

Data collection was performed in October 2020 through Amazon’s Mechanical Turk (MTurk). Previous studies have provided strong evidence to show that MTurk is a suitable survey tool to collect individual-level data [53]. According to Behrend et al [54], subjects recruited using MTurk are more representative of the US population in terms of age, gender, race, and work experience. Moreover, data collected through MTurk have been reported to be more reliable than those obtained through traditional data collection means, and meet the standards of social behavior studies [55]. Researchers as requesters can use this crowdsourcing website to reach out to potential subjects (ie, MTurk workers) in numerous countries to conduct a survey.

Several studies have compared MTurk to conventional data collection methods in the health and medical literature. The vast majority of these studies support the use of MTurk for a variety of academic purposes (eg, in health care research) [56]. Existing literature in clinical research highlights that due to a large number of users, the MTurk population is more representative of the US population at large than other online surveys [57]. In this study, the respondents’ location was limited to the United States. The incentive for participation was a monetary reward (US $1). The average completion time for the three groups was 4.3 minutes, which implied acceptable responses in terms of the time spent by respondents to complete the survey. Initially, 395 respondents attempted the survey.

As reported in previous studies, one main concern in online data collection is that subjects choose answers randomly or participate with less attention [58]. Therefore, two filters were used in this study to enhance the quality of the collected data. First, participation was restricted to MTurk workers with a high reputation (at least 80% approval ratings). Second, another solution for detecting careless, rushed, or haphazard answers in behavioral research is using “captcha” questions [59]. Thus, attention check questions were used to identify and exclude responses of participants who simply picked an answer choice without reading the questions or did not correctly answer reverse-coded filler items [60]. Insufficient answers and dropped responses that failed the response quality questions were removed. After removing unsatisfactory answers (31 data points), the final set of valid and useable responses included 364 samples.

### Instrument Validation

Confirmatory factor analysis was performed using IBM SPSS AMOS (Version 22) to find evidence for convergent validity and discriminant validity. The results of model fit indices for the measurement model demonstrated a good fit (goodness of fit index χ^2^_2.92_=0.83, adjusted goodness of fit index=0.80, comparative fit index=0.90, normed-fit index=0.91, incremental fit index=0.90, standardized root mean square residual=0.02, and root mean square error of approximation=0.03), all meeting their respective common acceptance levels.

Multicollinearity was checked by computing the variance inflation factor (VIF). The VIF values ranged between 1.218 and 1.551, which were below the cut-off value of 5 [52]. This confirmed that multicollinearity was not an issue in this study. Furthermore, because using a self-report survey can cause the common method variance issue, the potential for common method bias was carefully examined [61]. The Harman one-factor test was performed to check if the common method bias would be a considerable problem [62]. All factors together could explain 74.231% of the total variance, whereas none of the factors accounted for most of the covariance among measures (<20%). Thus, the test results indicated that common method bias is not a significant threat in the study sample.

According to Gefen et al [63], convergent validity can be determined by examining measures such as standardized factor loading, composite reliability, and the average variance extracted (AVE). The convergent validity test results are displayed in Table 2. The composite reliability values for all of the constructs in the model were above the threshold acceptance value of 0.7, highlighting the adequate reliability of constructs [64]. The reported standardized factor loadings for all of the constructs were found to be greater than 0.7, and according to Hair et al [65], a factor loading of 0.7 or greater is acceptable. The AVE was calculated using the standardized factor loading values for each of the constructs. All reported values of AVE were greater than 0.5, which is the minimum acceptable value [66]. These measures provided evidence that the convergent validity of the measurement model was acceptable. As the instrument validation results were satisfactory, no items were excluded from further analysis.

Discriminant validity analysis of the constructs was also performed. In Table 3, the main diagonal elements, representing a construct with itself, indicate the square roots of the AVEs, and the off-diagonal values represent the correlation coefficients between the constructs. All diagonal values were higher than 0.70 and exceeded correlations between any pair of constructs [67]. Therefore, the model fulfills the discriminant validity requirements, and it was assumed that the model also has adequate discriminant validity.

**Table 2:** Results of convergent validity.

Construct				Standardized factor loading (>0.70)		Composite reliability (>0.70)		AVE^a^ (>0.50)
**IT identity**								
	**Relatedness**					0.914		0.679
		REL1	0.82					
		REL2	0.80					
		REL3	0.83					
		REL4	0.86					
		REL5	0.81					
	**Emotional energy**					0.921		0.699
		EMO1	0.83					
		EMO2	0.85					
		EMO3	0.84					
		EMO4	0.82					
		EMO5	0.84					
	**Dependence**					0.915		0.682
		DEP1	0.82					
		DEP2	0.81					
		DEP3	0.82					
		DEP4	0.85					
		DEP5	0.83					
**Perceived gamification mechanism**						0.907		0.619
		PEG1	0.80					
		PEG2	0.77					
		PEG3	0.79					
		PEG4	0.79					
		PEG5	0.81					
		PEG6	0.76					
**Continued intention to use the app**						0.875		0.638
		CIU1	0.73					
		CIU2	0.81					
		CIU3	0.83					
		CIU4	0.82					
**Information-sharing tendency**						0.875		0.636
		IST1	0.72					
		IST2	0.72					
		IST3	0.78					
		IST4	0.80					

^a^AVE: average variance extracted.

**Table 3:** Results of the discriminant validity test.^a^

Construct	Mean (SD)	ITI-REL^b^	ITI-EMO^c^	ITI-DEP^d^	PEG^e^	CIU^f^	IST^g^
ITI-REL	3.664 (0.890)	*0.824*	0.514	0.523	0.311	0.371	0.338
ITI-EMO	3.865 (0.823)	0.514	*0.836*	0.598	0.369	0.114	0.319
ITI-DEP	3.937 (0.798)	0.523	0.598	*0.825*	0.301	0.125	0.248
PEG	3.639 (0.904)	0.311	0.369	0.301	*0.786*	0.431	0.374
CIU	3.938 (0.889)	0.371	0.114	0.125	0.431	*0.798*	0.372
IST	3.820 (0.853)	0.338	0.319	0.248	0.374	0.372	*0.797*

^a^Diagonals in italics (construct compared with itself) represent the square root of the average variance extracted; off-diagonals are the correlation values.

^b^ITI-REL: information technology identity-relatedness.

^c^ITI-EMO: information technology identity-emotional energy.

^d^ITI-DEP: information technology identity-dependence.

^e^PEG: perceived gamification mechanism.

^f^CIU: continued intention to use the app.

^g^IST: information-sharing tendency.

## Results

### Respondent Characteristics

Table 4 shows the participants’ characteristics. The descriptive statistics were calculated with IBM SPSS version 27. The most frequent apps mentioned by respondents were Fitocracy, Fooducate, MyFitnessPal, My Diet Coach, RunKeeper, Strava, and JEFIT Workout. Slightly less than half of the respondents reported that they have been using the app for 6 months to 1 year, and slightly more than half of the respondents indicated that they used the app on a daily basis. The majority of participants (78%) stated that they were either “extremely experienced” or “very experienced” with the app they mentioned in the survey. The demographic data demonstrated that the respondents were fairly equally distributed in terms of gender. Age range and income were normally scattered, with age range between 30 and 39 years and an annual household income between US $50,000 and $74,999 showing higher ranges among the provided categories. The majority of respondents were white, followed by African American and Hispanic. The majority of respondents had a full-time job. The most common education level was a bachelor's degree, followed by some college degree.

Regarding experience with general technology, the respondents were mostly familiar with mobile devices, with the majority (92%) rating themselves as either “extremely” or “very” familiar with mobile devices (such as phones and tablets). Concerning familiarity with health care technologies, approximately 73% of the respondents reported that they were either “extremely” or “very” familiar with mHealth apps. Finally, the majority of respondents participated in an online health community (eg, information sharing or posting comments).

**Table 4:** Sample characteristics (N=364).

Variable		Participants, n (%)
**Gender**		
	Male	204 (56.0)
	Female	160 (44.0)
**Age (years)**		
	<20	15 (4.1)
	20-29	84 (23.1)
	30-39	146 (40.1)
	40-49	66 (18.1)
	50-59	33 (9.1)
	≥60	22 (6.0)
**Annual household income (US $)**		
	<25,000	55 (15.1)
	25,000-49,000	76 (20.9)
	50,000-74,999	124 (34.1)
	75,000-99,999	58 (15.9)
	100,000-150,000	36 (10.0)
	>150,000	15 (4.1)
**Education**		
	Less than high school	4 (1.1)
	High school graduate	33 (9.1)
	Some college	95 (26.1)
	2-year degree	40 (11.0)
	Bachelor’s degree	135 (37.1)
	Master’s degree	47 (12.9)
	Doctorate	11 (3.0)
**Employment status**		
	Employed full time	251 (69.0)
	Employed part time	51 (14.0)
	Unemployed	40 (11.0)
	Retired	15 (4.1)
	Student	7 (1.9)
**Race/ethnicity**		
	White	189 (51.9)
	African American	84 (23.1)
	Asian	22 (6.0)
	Hispanic	58 (15.9)
	Mixed	7 (1.9)
	Other	4 (1.1)
**Apps used**		
	Fitocracy	142 (39.0)
	Fooducate	66 (18.1)
	MyFitnessPal	51 (14.0)
	My Diet Coach	44 (12.1)
	RunKeeper	33 (9.1)
	Strava	18 (4.9)
	JEFIT Workout	11 (3.0)
**How long have you used the health app?**		
	Less than 6 months	36 (9.9)
	6 months to 1 year	167 (45.9)
	1-2 years	135 (37.1)
	More than 2 years	25 (6.9)
**How often do you use the health app?**		
	Daily	186 (51.1)
	Weekly	138 (37.9)
	Monthly	40 (11.0)
**To what extent are you experienced with the particular health app you use?**		
	Extremely experienced	153 (42.0)
	Very experienced	131 (36.0)
	Moderately experienced	66 (18.1)
	Slightly experienced	15 (4.1)
**To what extent are you familiar with mobile devices (eg, phones, tablets)?**		
	Extremely familiar	244 (67.0)
	Very familiar	91 (25.0)
	Moderately familiar	25 (6.9)
	Slightly familiar	4 (1.1)
**To what extent are you generally familiar with mobile health apps?**		
	Extremely familiar	153 (42.0)
	Very familiar	113 (31.0)
	Moderately familiar	66 (18.1)
	Slightly familiar	33 (9.1)
**Have you ever participated in an online health community (such as information sharing or posting comments)?**		
	Yes	244 (67.0)
	No	120 (33.0)

### Structural Model

**Figure 2:**
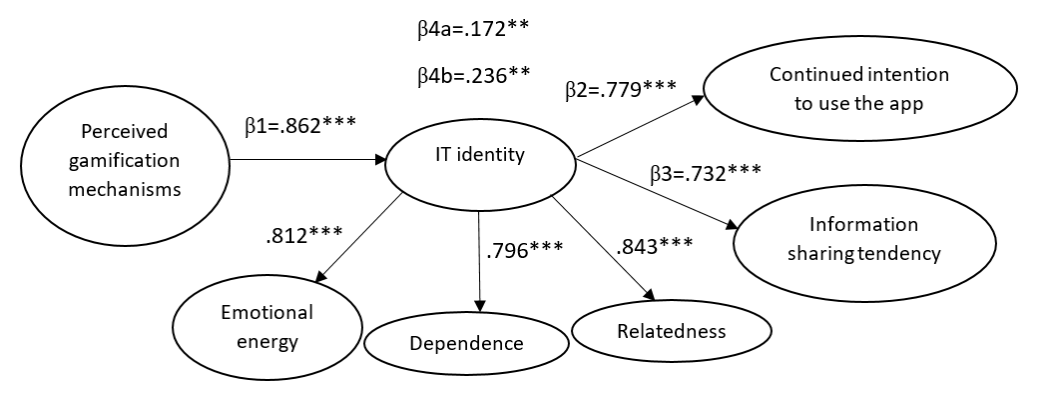
Model paths. IT: information technology. **P<.01, ***P<.001.

IBM SPSS AMOS (Version 22) was used to test the hypotheses within a structural equation modeling framework. According to Ho [68], the goodness of fit statistics can access the entire structural model and measure the overall fit. The normed χ^2^ value was 2.03, which was between the recommended values of 1 and 3 [69]. The values for the comparative fit index (0.914), normed fit index (0.926), relative fit index (0.912), and Tucker-Lewis index (0.903) were above 0.9, and the standardized root mean square residual (0.034) and root mean square error of approximation (0.058) were below 0.08 [70]. The value of the adjusted goodness of fit index was 0.921, which exceeded the threshold of 0.90. All mentioned measures of fit were in the acceptable range, and only the goodness of fit index (0.837) was marginal. Based on Kline [71], at least four statistical indices should meet the minimum recommended values. More than four indices satisfied the cut-off values in this study, which supported a good fit between the hypothesized model and observed data. Figure 2 exhibits the standardized path coefficients of the structural model.

Consistent with a previous study [18], IT identity was measured using three interrelated dimensions (relatedness, emotional energy, and dependence). In a reflective construct, dimensions have positive and significant intercorrelations as they share the same pattern [72]. The loadings (β values) were .812 for emotional energy, .796 for dependence, and .843 for relatedness. The results imply that the three dimensions of IT identity as first-order factors load significantly on the second-order construct. Thus, the combination of three dimensions reflects IT identity in relation to health or fitness apps.

Path coefficients were examined to assess the structural model. The results of the hypotheses testing are summarized in Table 5. The findings support H1 by showing that perceived gamification mechanisms significantly enact IT identity. H2 was also supported in which a higher IT identity in relation to health or fitness apps significantly shapes continued intention to use them. The findings also provided sufficient evidence to support H3, which indicates that users with a stronger IT identity in relation to health or fitness apps are more likely to share their personal health information via the apps. The indirect effect of perceived gamification mechanisms on continued intention to use through enacted IT identity was significant. However, the direct relationship between perceived gamification mechanisms and continued intention to use was not significant (β=.031, *P*=.12). Thus, it can be concluded that the impact of gamification mechanisms on continued intention is fully mediated by IT identity, supporting H4a. The analysis also demonstrated that perceived gamification mechanisms indirectly help build information-sharing tendency through activated IT identity. Nevertheless, there was no significant direct relationship between perceived gamification mechanisms and information-sharing tendency (β=.071, *P*=.10). Therefore, there is sufficient evidence to conclude that IT identity fully mediates the relationship between perceived gamification mechanisms and information-sharing tendency, supporting H4b.

Finally, the model explained 74% of the variance in IT identity, 60% of the variance in continued intention to use the app, and 53% of the variance in the information-sharing tendency. These R^2^ values suggested that the model provides relatively strong explanatory power to predict the variance in the positive use behaviors associated with health and fitness apps.

**Table 5:** Structural equation modeling results.

Hypothesis	Path	Standardized β coefficient (SE)	*P* value	Critical ratio	Results
H1	PEG^a^ to ITI^b^	.862 (.078)	<.001	11.160	Supported
H2	ITI to CIU^c^	.779 (.068)	<.001	10.812	Supported
H3	ITI to IST^d^	.732 (.073)	<.001	10.847	Supported
H4a	PEG to ITI to CIU (Mediating role of ITI)	.172 (.081)	.009	2.625	Supported
H4b	PEG to ITI to IST (Mediating role of ITI)	.236 (.094)	.003	2.471	Supported

^a^PEG: perceived gamification mechanism.

^b^ITI: information technology identity.

^c^CIU: continued intention to use the app.

^d^IST: information-sharing tendency.

## Discussion

### Principal Findings

Many companies and vendors have developed various mHealth apps with the use of gamification principles. Building a gamified health and fitness app requires substantial investment. However, a highly competitive market and misalignment of gamification mechanisms with main users’ motivations could result in switching or discontinuance uses [47]. According to Conroy et al [73], many fitness apps use similar designs with ineffective gamification techniques. Moreover, few studies have been performed to identify how the best gamification mechanisms should be used in mHealth apps [11]. This study helps both theory and practice to build a better understanding of the relationships among gamification, IT identity, and positive use behaviors (such as continued usage and information-sharing tendency). This study has implications for theory building on IT identity in the context of health and fitness apps. Without a clear picture of beliefs associated with the three dimensions of IT identity (ie, emotional energy, dependence, and relatedness), postadoption behaviors may be compromised. Therefore, the IT identity concept can be integrated into the postadoption research area, as it can help researchers recognize why individuals use health and fitness apps in their own ways. The results can confirm previous studies explaining why people try to maintain consistency between identities and behaviors [74].

This study further contributes to knowledge by shedding light on how gamification mechanisms will translate into positive use behaviors for regular users of mHealth apps. The findings highlight that gamification itself does not necessarily encourage users to share their personal data and keep up with health and fitness apps. Based on the fully mediating role of IT identity, gamified designs will be a successful technique to encourage meaningful interactions with the apps only if gamified elements can enact users’ emotional responses to thinking about themselves in relation to health and fitness apps. This could be a plausible reason for the insufficient effectiveness in the design of many gamified health and fitness apps to attract and keep their users. Previous studies have mentioned that infective gamification may lead to the users’ discontinuance intention in the context of health and fitness apps [47]. This study provides a richer explanation and defines ineffective gamification as gamified elements that cannot contribute to individuals’ self-identification. The main theoretical contribution of this study is to determine the missing link between gamification and positive use behaviors. Based on identity theories, it is proposed that IT identity is an overlooked factor that can bridge the effects of gamification to positive use behaviors. According to this logic, if gamified features of health and fitness apps cannot generate a sense of emotional energy, dependence, and relatedness to the apps, current users are more likely to discontinue interacting with them after a few months. Using the same reasoning, gamification that is not coupled with IT identity may not motivate existing users to have more interactions with the apps by sharing their personal health information.

Previous studies indicate that gamification directly influences personal information disclosure [75]. Individuals may use a gamified fitness app to share their experiences and information with friends, and gain social value. This study extends this point by incorporating the IT identity view to examine information-sharing willingness triggered by self-identification with the apps. Individuals may use gamified elements to generate a sense of challenge and compete with other users. This feeling gives rise to a sense of need and enjoyment with the apps. To stay in the social engagement loops and participate in social interactions with others, they saliently rely and depend on their apps. Thus, the use of the apps will be integral to their sense of self to exhibit how they are in completing challenges and quests. This strong IT identity represents positive self-identification, and motivates them to share more personal information and stay highly engaged with the apps.

Focusing on pure gamification mechanisms to increase market share can be a quick fix for the designers of mHealth apps. Using various gamified elements (such as badges, leaderboards, and points) can inspire individuals to use the apps, but they cannot automatically motivate them to stay with the apps. This is consistent with previous studies suggesting that gamified designs (such as using badges) should not be used alone because users may not be excited enough to continue to use the apps without further motivation and reinforcement [9]. This study proposes that continuous and enhanced use of health and fitness apps will need another source of motivation rooted in emotion, positive energy, enthusiasm, and a strong sense of reliance and connection with the apps. Gamified elements should be meaningful if users are awarded for achieving specific goals appropriate for the apps.

This finding could be a practical contribution to mHealth designers by demonstrating that gamified features that are not consistent with the apps’ main purposes will not act as a meaningful incentive. This is in line with previous studies highlighting that putting too much emphasis on gamification mechanisms may negatively affect users’ experience with the apps [76]. A suitable blend of gamification designs that help users complete defined tasks should foster IT identity by connecting app usage with users’ habits. If users find the combination of gamified elements useful in accomplishing goals, they feel a strong sense of competence and enthusiasm. Thus, to enact these positive feelings and satisfy the experienced emotion, they are more likely to stay with the app and use it for additional health-related tasks. The activated IT identity may then inspire current users to think of themselves in relation to the apps on a daily basis and provides a basis for understanding the self in relation to IT.

Robust gamification mechanisms will help shape IT identity only when an individual has confidence in using their app to complete various tasks in different situations that can actualize intrinsic benefits and emotional rewards. The findings imply that effective rewards and incentives (eg, points and badges) accessible through gamified health and fitness apps should first create durable enjoyment, reliance, and connection with the apps. Otherwise, these gamified features may not motivate users to identify the self with the apps. If users do not consider the apps as an integral part of the self (due to lack of emotional responses), they may use the apps for a while but are very likely to switch to other alternatives supporting their self-concept. The results suggest that app designers may not increase feature use behaviors and enhanced use behaviors through gamification alone, but use behaviors can be improved through positive self-identification. Therefore, gamified incentives that foster self-identification by creating self-confirming sense, positive energy, and emotion associated with mHealth apps are more likely to encourage current users to keep up with them. Effective gamification mechanisms accompanied by a strong IT identity can reinforce health and fitness apps’ usability, encourage continued use, and inspire patient self-management.

Finally, these findings have important practical implications for those tasked with the responsibility of developing health and fitness apps. There are several active alternatives and a wide range of health and fitness apps on the mHealth market [77]. Thus, developers and vendors of such apps should pay close attention to the importance of the IT identity verification process if they desire to engage users and promote positive postadoption behaviors. The absence of emotional energy, reliance, and connection between users and health apps may create negative feelings such as boredom and lack of interest. Considering IT identity, vendors should make an effort to understand its three dimensions (ie, dependence, relatedness, and emotional energy) and how to support each of them through effective gamification mechanisms. Vendors must have a thorough understanding of what combination of gamified elements (eg, badges, leaderboards, points and levels, challenges, and social engagement) can contribute to the verification of IT identity in relation to health and fitness apps. Moreover, vendors need to consider the indirect role of gamification to encourage continued intention and information-sharing tendency. By examining IT identity and its relationships with active use behaviors, vendors and developers can be provided with practical recommendations about improving users’ value derived from mHealth apps and retaining as well as engaging users.

### Limitations and Future Study

First, in this study, data were collected from a sample of respondents from the United States. The culture of using health and fitness apps is diverse among different countries. Therefore, caution should be exercised when generalizing the results. It is recommended that future studies consider subjects from other geographical locations such as other developed countries and developing countries with different technology infrastructure.

Second, it is important to mention that a general limitation with cross-sectional or short-term gamification studies is the study duration. Thus, longitudinal studies can provide a clearer understanding of the long-term effects of gamification on behavior outcome, user engagement, and continued use behavior.

Third, this study used a self-rated sample through an online survey to recruit participants digitally. Although several measures were taken to provide clear definitions, there is still a small chance that some respondents were not completely aware of the gamification mechanisms and may have formed their own perceptions of the IT artifact. For this reason, perceptions (perceived gamification mechanisms) were included in the research model to tackle this issue. Further studies should use an alternative method (eg, experiment) to ensure that subjects are knowledgeable about gamification to measure this construct more accurately.

Fourth, current users of gamified health and fitness apps were recruited for this study; focusing on a population of engaged users can also limit the generalizability of the findings. The role of IT identity may apply to this population but may be different in nonapp users. Thus, these findings are applicable among regular users of mHealth apps. This study calls for more research to improve generalizability by using a more comprehensive user status (such as current users, potential users, previous users, and nonusers).

Fifth, data were collected from respondents of some gamified health and fitness apps. All of the mentioned apps shared a combination of the most commonly used gamification mechanisms in mHealth apps (such as badges, challenges, points, levels, and feedback), which can satisfy this study’s objectives. As suggested previously [11], gamification is considered a collection of multiple conditions, and none of these conditions alone is sufficient to constitute a gamified service. However, it should be acknowledged that their designs and goals may be different, and there may be a different effect with varying gamification strategies. Therefore, future research is required to further analyze how different aspects of gamification may affect users’ perceptions. Moreover, future research can examine the proposed model by focusing on only one gamified app.

Sixth, using an online survey may generate a sample selection bias. Data were collected only from people who could access a computer, mobile devices, and the internet to participate in the online survey. Future studies can use other data collection means and sampling strategies to recruit a sample that is generalizable to a wide range of health care consumers. Seventh, this study did not focus on a specific brand of health and fitness app. It would be interesting to examine whether alternative mHealth brands could influence verification of IT identity and, in turn, shape positive use behaviors.

Finally, consistent with this study’s purpose, the main goal was to develop and test a research model centered on the IT identity concept. Thus, many other widely used constructs from technology adoption models were not included in the research model. Future studies can incorporate other constructs that may enhance the amount of variance in IT identities, such as social influence, performance expectancy, and effort expectancy. Further research can also use objective measures to analyze the continued intention and positive use behaviors, such as usage frequency or number and the recency of sharing personal information via health and fitness apps.

### Conclusions

Given the increasing importance of gamification and its impacts on the continuance of usage among regular users of health and fitness apps, this study proposes a model centered on the IT identity lens to fill current research gaps. Based on IT identity theories, it is proposed that effective gamification mechanisms embedded in health and fitness apps can activate users’ IT identity and improve postadoption behaviors. The results suggest that an appropriate mix of gamified elements helps users emotionally connect to health and fitness apps and have more control over their feature set. These feelings foster their self-identification in relation to the apps, and in turn, they will be more likely to engage in information sharing and enhance use behaviors. Based on the results of this empirical study, it is proposed that IT identity fully mediates the effects of gamification on positive use behaviors. On the same basis, only gamified elements that effectively activate individuals’ IT identity will result in continuous interactions with the apps and enhanced information sharing. Gamified features that better communicate the purpose of a health app and help users identify themselves with the app are more likely to foster active information sharing and continued intention to use. The findings propose theoretical implications to the gamification literature by demonstrating the mediating role of IT identity in shaping positive use behaviors. This study contributes more in-depth knowledge to the gamification principles in the context of mHealth apps and provides useful insights into the design of an effective gamified application platform. With a deeper understanding of IT identity and its relationship with gamification, mHealth app developers may be better positioned to design gamified features supportive of IT identity to improve postadoption use behaviors.

## Appendix

Multimedia Appendix 1 Online survey.

## Abbreviations

AVE: average variance extracted
IT: information technology
mHealth: mobile health
MTurk: Amazon's Mechanical Turk
VIF: variance inflation factor
