# Supplementary material for: The Influence of Gamification and Information Technology Identity on Postadoption Behaviors of Health and Fitness App Users: Empirical Study in the United States
Source: JMIR Serious Games. 2021 Jul 5;9(3):e28282. doi: 10.2196/28282 (PMC8406121; doi:10.2196/28282)
Supplement: Multimedia Appendix 1 [file games_v9i3e28282_app1.docx]

**Appendix – Online survey**

| Construct | Subdimensions | Items | Questions |
| --- | --- | --- | --- |
| IT identity | Relatedness  Emotional Energy  Dependence | REL1  REL2  REL3  REL4  REL5  EMO1  EMO2  EMO3  EMO4  EMO5  DEP1  DEP2  DEP3  DEP4  DEP5 | I express feelings of connectedness when thinking of myself in relation to the health app  Thinking of myself in relation to the health app, I feel that I am close with the device  Thinking of myself in relation to the health app, I feel a strong sense of connection with the device  Thinking of myself in relation to the health app, I see myself linked with the device  Overall, thinking of myself in relation to the health app, I feel that my relatedness to the device to manage my health is high  Thinking of myself in relation to the health app, I feel an emotional attachment to the device  Thinking of myself in relation to the health app, I feel enduring enthusiasm about the device  Thinking of myself in relation to the health app, I express feelings of confidence in the device  Thinking of myself in relation to the health app, I express feelings of energy  Overall, thinking of myself in relation to the health app, I feel that my emotional energy levels to the device are high  I express feelings of reliance when thinking of myself in relation to the health app  Thinking of myself in relation to the health app, I feel a sense of dependence upon the device to manage my healthcare  Thinking of myself in relation to the health app, I feel that I can count on the device to monitor my health information  Thinking of myself in relation to the health app, I feel that I need the device to control my health status  Overall, thinking of myself in relation to the health app, I feel that my sense of reliance on the device is high |
| Perceived gamification mechanism | N/A | PEG1  PEG2  PEG3  PEG4  PEG5  PEG6 | I think that the health app has badges to identify and reward individual achievements.  I believe the health app has leaderboards to rank individual user progress and achievements as compared to other peers  I think that the health app has a points and levels mechanism to earn points for completing different levels  I believe that the health app has challenges and quests through competition  I think that the health app tells me what I have accomplished lately through feedback  I believe the health app has social engagement loops through social media profiles |
| Continued intention to use the app | N/A | CIU1  CIU2  CIU3  CIU4 | I intend to continue using the health app.  I want to continue using the health app rather than discontinue it.  I predict I will continue using the health app.  I plan to continue using the health app. |
| Information sharing tendency | N/A | IST1  IST2  IST3  IST4 | I am very likely to reveal my health information in the health app  I am willing to disclose my health information to others through the health app  In the future, I am willing to provide my personal information to others who use the health app  I will probably release my health information to the health app |

ALL items were measured on a 1 = Strongly Disagree, 5 = Strongly Agree Likert scale.
